# Diagnostic significance of gut Microbiome dysbiosis and biomarker expression in Egyptians with hepatocellular carcinoma

**DOI:** 10.1038/s41598-025-28663-6

**Published:** 2025-12-01

**Authors:** Amani E. Marawan, Omar Ahmed Elmetwally, Mohamed M. Marwan, Mohamed M. A. El-Sokkary, Shimaa A. Abass, Laila A. Eissa

**Affiliations:** 1 Faculty of Veterinary Medicine, Delta University for Science and Technology, Gamasa, 11152 Egypt; 2 Shoha Veterinary Teaching Hospital, Faculty of Veterinary Medicine, Mansoura University, Mansoura, 35738 Egypt; 3 Internal Medicine Department, Faculty of Medicine, Mansoura University, Mansoura, Egypt; 4 Department of Pharmacology and Biochemistry, Faculty of Pharmacy, Horus University, New Damietta, 34518 Egypt; 5 Microbiology and Immunology Department, Faculty of Pharmacy, Mansoura University, Mansoura, 35516 Egypt; 6 Biochemistry Department, Faculty of Pharmacy, Kafreksheikh University, Kafreksheikh, 33516 Egypt; 7 Department of Biochemistry, Faculty of Pharmacy, Mansoura University, Mansoura, 35516 Egypt

**Keywords:** Hepatocellular carcinoma, Gut microbiome, Biomarkers, VEGF, And MMP9., Biochemistry, Cancer

## Abstract

Egypt has the greatest incidence of hepatitis C virus (HCV) infection globally, which is a considerable trigger of fibrosis, cirrhosis, and hepatocellular carcinoma (HCC). The gut microbiome has been recognized for contributing to various hepatic conditions. Nevertheless, its correlation with HCV and HCC is not well understood. Our study is conducted to investigate the potential relevance of some biomarkers: matrix metalloproteinase 9 (MMP9), Signal transducer and activator of transcription 3 (STAT3), superoxide dismutase (SOD), vascular endothelial growth factor (VEGF), and nuclear factor-κB (NF-κB), along with gut microbial variations, for the differentiation between HCV-related cirrhosis and HCC. The 90 fecal and blood samples were collected from 30 healthy controls, 30 HCV-related cirrhosis, and 30 HCC for detecting gut microbial abundance and biochemical markers examination. Our findings displayed the existence of intestinal microbiome dysbiosis with marked enrichment of specific species, including *Bifidobacterium*,* Fusobacterium*, *Providencia*,* E. faecium*, and *Pseudomonas aeruginosa* in HCC patients. Furthermore, the most enriched genera in HCV-related cirrhosis patients were *Bifidobacterium*, *Porphyromonas*, and *Bacteroides*. MMP9 exhibited the highest diagnostic performance of the five measured biomarkers, discriminating against HCC vs. HCV-related cirrhosis with specificity and sensitivity of 100% and 90%, respectively, at a cut-off value > 166.8. Additionally, SOD and NF-κB were statistically significant discriminators of HCC from cirrhosis at cutoff values of ≤ 0.197 and > 166.8. A significant correlation between microbiome abundance and VEGF and MMP9 was observed. This study illustrated that gut microbiomes contribute to HCC and HCV-related cirrhosis pathogenesis, opening approaches for cancer management and prevention.

## Introduction

Hepatocellular carcinoma constitutes approximately 80% of liver cancer. It is the sole significant malignancy for which mortality rates have remained stagnant over the past decade^1^. The HCC prognosis is unfavorable, with a five-year survival rate under 20%^2^. HCC arises from post-hepatitis C virus (HCV), chronic hepatitis B virus (HBV) infection, tobacco use, cirrhosis, aflatoxin exposure, and prolonged alcohol use. Viral hepatitis is categorized as the seventh main cause of mortality globally^3^. In Egypt, HCV infection, mostly of genotype 4^4^ constitutes a public health issue having the greatest prevalence across countries, as reported by the WHO^5^. Post-viral hepatitis HCC mostly arises from oxidative stress, hepatic inflammation, oncogene activation, dysregulated signaling pathways, and the integration of viral nucleic acid into the host genome^6^.

The stealthy progression of HCC, coupled with the absence of reliable biomarkers for its detection, complicates its early diagnosis when treatment is most efficacious. The sole curative method for HCC is surgical resection, which is beneficial exclusively to patients having localized tumors^7^. Consequently, it is imperative to discover novel HCC biomarkers and therapeutic targets to enhance its diagnosis and treatment. A recent study highlights the gut microbiome’s influence on immune system modulation and its association with diverse disease states^8^. The gut microbiome comprises a diverse range of gastrointestinal bacteria, affected by environment, diet, age, host genetics, and pharmacological therapies^9,10^. The variety and equilibrium of the ecology of these microbes are crucial for sustaining host health^11^.

The liver, due to its significant immunoregulatory function, metabolic role, and regeneration ability, maintains a considerable connection with the gut through enterohepatic circulation^12^. It was reported that the imbalance of the gut microbiome contributes to HCC development^12,13^.The gut microbiome induces hepatocarcinogenesis through several pathways, as microbiota dysbiosis enhances bacterial translocation, exacerbating liver inflammation. Additionally, bacterial overgrowth generates substantial quantities of lipopolysaccharides, which engage the macrophages’ toll-like receptors, resulting in their activation. Activated macrophages secrete specific proinflammatory cytokines (tumor necrosis factor (TNF), interleukin 17 (IL-17), and IL-6) and activate signaling pathways (signal transducer and activator of transcription 3 (STAT3) and nuclear factor-κB (NF-κB)), increasing the HCC cells’ growth and inhibiting their apoptosis. Finally, genotoxins generated by the microbiota are transported to the liver cell nucleus, damaging the DNA^14^.

Dysbiosis of gut microbiomes logically exacerbates the pathogenesis of viral hepatitis by provoking inflammatory cascades, leading to HCC development^15^. The mechanisms by which viral hepatitis disrupts gut microbiota remain unidentified; nonetheless, preserving gut homeostasis may avert the advancement of hepatic illness and the development of HCV-mediated HCC^5^. Although alpha-fetoprotein (AFP) is commonly used for HCC diagnosis, its limitations in sensitivity and specificity, particularly in early-stage disease^16^, necessitate alternative or complementary biomarkers. Therefore, in this study, we selected STAT3, NF-κB, SOD, VEGF, and MMP9 due to their established involvement in cancer-related pathways such as inflammation, oxidative stress, angiogenesis, and metastasis. These biomarkers were investigated for their potential diagnostic relevance and their correlation with microbial dysbiosis in differentiating HCV-related cirrhosis from HCC.

## Materials and methods

### Study design and participants

This *case-control* study included 90 participants and was conducted from March to September 2023 at Mansoura University Hospital (Departments of Internal Medicine and Gastroenterology), in collaboration with the Biochemistry Department, Faculty of Pharmacy, Mansoura University, Egypt. Participants were divided into three age- and sex-matched groups: 30 patients with hepatocellular carcinoma (HCC), 30 with hepatitis C virus (HCV)-related cirrhosis, and 30 healthy controls. HCC and HCV-related cirrhosis cases were identified through clinical assessment and confirmed using imaging (triphasic abdominal computed tomography, TACT), serological markers, and biochemical analyses^17^. HCC diagnosis was supported by elevated serum alpha-fetoprotein (AFP) levels. Chronic HCV infection was confirmed by positive HCV RNA PCR without evidence of malignancy. Healthy controls were recruited from hospital staff and were confirmed to have no history or clinical signs of liver disease, negative viral hepatitis markers, normal liver function tests, and unremarkable abdominal ultrasounds. Inclusion criteria required participants to be adults (≥ 18 years). HCC patients had confirmed diagnoses based on clinical, imaging, and laboratory data. HCV patients had chronic infection without signs of carcinoma. Controls were healthy individuals free of hepatic pathology. Exclusion criteria included age under 18, recent antibiotic use (within two weeks), autoimmune hepatitis, HIV or HBV co-infection, excessive alcohol intake, and other known causes of liver disease. Comprehensive medical histories, including prior antiviral treatments, were obtained. All participants underwent thorough clinical evaluation, laboratory testing, and imaging as appropriate. This study was approved by the Ethical Committee of the Faculty of Medicine, Mansoura University (Approval Code: R.23.06.2237), in accordance with the declaration of Helsinki, and all participants provided written informed consent.

### Sample size calculation

#### Hypothesis

##### Null hypothesis: 

No statistically significant difference in inflammatory markers and microbiota distribution among the three study groups.

##### Alternative hypothesis:

A statistically significant difference in inflammatory markers and microbiota distribution between the three study groups with a large effect size (Cohen’s f = 0.4). This hypothesis was according to a previous study^18^.

##### Sample size:

G*Power software for Windows (version 3.1.9.7) was utilized to calculate the sample size. In a one-way ANOVA study, sample sizes of 30 participants per group were collected from each of the three groups, where their means were compared. The sample of 90 participants attains 92.6% power to identify variations among the means compared to the alternative of equal means, utilizing an F test at a significance level of 0.05. The magnitude of the variance in the means is denoted by the effect size f = σm/σ, which equals 0.40.

### Blood samples and biochemical assays

Under sterile conditions, 10 ml blood specimens were gathered from each participant for further conducting the routine laboratory tests. These included liver function tests (alanine aminotransferase (ALT), aspartate aminotransferase (AST), albumin, and total bilirubin), serum creatinine, complete blood counts using Sysmex, international normalized ratio (INR), and prothrombin concentration. HCV polymerase chain reaction (PCR) evaluation was conducted utilizing reverse transcription PCR (Roche COBAS TaqMan HCV assay version 2.0, with a minimum detecting limit of 15 IU/ml). The estimation of Hepatitis B surface antigen and determination of HCV antibodies were conducted utilizing the automated MiniVidas immunoassay technology^19^.

### Real-time PCR test for detecting the relative expression of gut microbiomes

The real-time quantitative PCR technique was conducted to ascertain the relative expression of gut microbiomes, including *Bacteroides* spp., *Porphyromonas gingivalis*,* Bifidobacterium* spp., *Enterococcus faecium*, *Fusobacterium* spp., *Pseudomonas aeruginosa*,* Providencia* spp., and *Clostridium* spp. During medical examination, fresh stool samples were obtained from all applicants and instantly kept at − 80 °C. The QIAamp Fast DNA Stool Mini Kit (50) (LOT 163047148) was used for extracting microbial DNA, following the manufacturer’s guidelines. A NanoDrop device (OPTIZEN NanoQ, Mecasys) was used to measure the concentration of DNA spectrophotometrically. Then DNA samples were frozen at −80 °C for further analysis. SYBR Green PCR master mix (Willowfort Co., UK) was used for quantitative DNA measurements from isolated fecal DNA using each forward and reverse primer (Table 1). The following parameters were adjusted for all real-time reactions: 95 °C for 5 min, followed by 45 cycles of 95 °C for 20 s, an annealing temperature for 20 s (refer to Table 1), and 72 °C for 40 s. The RT-PCR reactions were conducted using the MyGo real-time PCR machine and its associated software. The Ct values were detected and melting curves were analyzed to ensure amplification specificity. The 2^ΔΔCt^ method was used to measure the relative abundance of each microbiome normalized to the total bacterial count in the corresponding specimen.

**Table 1:** The different primers used for detecting different bacterial species.

Primer name	Primer sequence		Annealing Tm	Size bp	Citation
*E. Faecium*	F	GCAAGGCTTCTTAGAGA	46.5	564	^20^
	R	CATCGTGTAAGCTAACTTC			
*Bifidobacterium*	F	CTCCTGGAAACGGGTGG	51	551	^21^
	R	GGTGTTCTTCCCGATATCTACA			
*Porphyromonas gingivalis*	F	AATCGTAACGGGCGACACAC	53	594	^22^
	R	GGGTTGCTCCTTCATCACAC			
*Fusobacterium*	F	GGATTTATTGGGCGTAAAGC	51.5	162	^23^
	R	GGCATTCCTACAAATATCTACGAA			
*Providencia*	F	ACCGCATAATCTCTTAGG	43.5	514	^24^
	R	CTACACATGGAATTCTAC			
*Clostridium* spp.	F	CGGTACCTGACTAAGAAGC	50	429	^25^
	R	AGTTTGATTCTTGCGAACG			
*P. aeruginosa*	F	CGAGTACAACATGGCTCTGG	53	116	^26^
	R	ACCGGACGCTCTTTACCATA			
*Bacteroides* sp.	F	AAGGGAGCGTAGATGGATGTTTA	55	193	^27^
	R	CGAGCCTCAATGTCAGTTGC			
*All bacteria*	F	GAGTTTGATCCTGGCTCAG	51	312	^28^
	R	GCTGCCTCCCGTAGGAGT			

### Assessment of the oxidative stress parameters

The liver’s antioxidant capacities were assessed by measuring the superoxide dismutase (SOD) based on the previously described method by Nishikimi et al^29^. The alteration in absorbance was observed at 420 nm.

### Gene expression analysis of blood biomarkers

The RNA was isolated from frozen blood samples utilizing an available RNA extraction kit (Direct-zol RNA MiniPrep, Zymo Research Co., U.S.A.). Firstly, the TRIzol reagent was utilized to obtain a high concentration. After that, the obtained supernatant was applied to a mini-spin column. The purity of RNA was determined by the A260/A280 ratio, with an acceptable range of 1.8 to 2.1. One µg of RNA from each sample was used to obtain complementary DNA according to the instructions of the Quantitect Reverse Transcription kit acquired from Qiagen, Valencia, USA. The primers used are listed in Table 2. Quantitative real-time PCR analysis was conducted utilizing cDNA samples and a Maxima SYBR Green/Fluorescein master mix (Fermentas, United States). 2^−ΔΔ C T^ method was exploited to detect the fold change of the expression of each mRNA corresponding to GAPDH.

**Table 2:** The forward and reverse primers of each gene.

Gene name	Primer sequence	References
STAT3	F-GCCAGAGAGCCAGGAGCA R-TGAAGCTGACCCAGGTAGCGCTGC	^30^
VEGF	F-5′-GCA CCC ATG GCA GAA GG-3′ R- 5′-CTC GAT TGG ATG GCA GTA GCT-3′	^31^
NF-κB	F-5′-CCA TGA CAG CAA ATC TCC-3′ R-5′-TAA ACT TCA TCT CCA CCC C-3′	^32^
MMP9	F-5′-GCCTTTGGACACGCACG-3′ R-5′-AGCGGTCCTGGCAGAAATAG-3′	^33^
GAPDH	F 5′-TGG TAT CGT GGA AGG ACT CAT-3′ R-5′-ATG CCA GTG AGC TTC CCG TTC AGC-3′	^32^

### Statistical analysis

The data were analyzed utilizing version 20 of the Statistical Package for the Social Sciences program (SPSS, Inc., Chicago, Illinois, USA). Qualitative data were expressed as percentages and frequency. Quantitative data were represented by range and median (for non-normally distributed data). The Fisher’s exact and χ2 tests were employed to compare categorical data between groups. The Mann–Whitney test was used to compare two groups, while the Kruskal–Wallis test was used to compare more than two groups. The odds ratio and 95% confidence interval derived from the logistic regression were utilized. The diagnostic performance was detected using the Receiver-operating characteristic curve analysis. A P value below 0.05 at a 95% confidence interval is supposed to be significant.

## Results

### Clinical characteristics of HCC patients

The clinical characteristics of all participants are summarized in Table 3. The control group included 30 individuals (15 males and 15 females), the HCC group comprised 30 individuals (22 males and 8 females), and the HCV-related cirrhosis group comprised 30 individuals (18 males and 12 females). The median age of the HCC group and HCV-related cirrhosis group is 62.26 and 57.86, respectively. Based on self-reporting, 6.7% of the HCC patients were diabetic, while only 3.3% suffered from hypothyroidism. 83.3% of the HCC group were infected with HCV. On the other hand, 33.3% of the HCV-related cirrhosis group were diabetic, and only 3.3% of the group suffered from hypertension or hypothyroidism.

The BCLC staging system was used to classify HCC patients according to tumor burden, liver function, and performance status. Most patients (83.3%) were classified as stage D, indicating advanced disease, while 16.7% were stage C. The Child-Pugh classification, used to assess hepatic functional reserve, showed that most HCV-related cirrhosis patients were Child-Pugh class A (86.7%), whereas most HCC patients were class B (63.3%) or class C (20%), reflecting impaired liver function in the HCC group. Additionally, the FIB-4 score, an established noninvasive fibrosis index, revealed that 70% of HCC patients and 63.3% of cirrhotic patients had advanced fibrosis (FIB-4 > 3.25), confirming a higher degree of hepatic injury in the HCC group.

**Table 3:** Socio-demographic characteristics of patients with HCC and HCV-related cirrhosis.

Variable	HCC group (*N* = 30)	HCV-related Cirrhosis (*N* = 30)
Age	62.26 ± 9.62	57.86 ± 8.012
Sex		
Male	22 (73.3%)	18 (60%)
Female	8 (26.7%)	12 (40s)
Comorbidity		
Diabetes	2 (6.7%)	10 (33.3%)
Hypertension	30 (100%)	1 (3.3%)
Hypothyroidism	1 (3.3%)	1 (3.3%)
HCV	25 (83.3%)	
AFP	383.71 ± 379.5	77.67 ± 226.7
Complete blood count		
Total leukocytic count	7.7 ± 3.79	8.11 ± 3.74
Hemoglobin level (g/dl)	11.023 ± 2.02	11.23 ± 2
Platelet	127.73 ± 58.38	132.86 ± 40.065
Liver function test		
ALT (IU/L)	104.86 ± 314.97	91.96 ± 221.162
AST (IU/L)	158.3 ± 497.64	181.26 ± 651.55
AST/ALT ratio (IU/L)	1.54 ± 0.809	1.69 ± 1.01
Serum albumin (g/dl)	2.883 ± 0.418	3.73 ± 0.671
Serum direct bilirubin (mg/dl)	1.816 ± 2.47	1.422 ± 2.58
Serum total bilirubin (mg/dl)	2.706 ± 3.716	2.21 ± 3.88
INR	1.44 ± 0.393	1.27 ± 0.38
Renal function test		
Serum creatinine (mg/dl)	1.45 ± 0.913	1.34 ± 0.998
BCLC Staging		
C	5 (16.7%)	
D	25 (83.3%)	
Child-Pugh class		
A	5 (16.7%)	26 (86.7%)
B	19 (63.3%)	
C	6 (20%)	4 (13.3%)
FIB4		
< 1.45 (Ishak fibrosis stages 0–1)	1 (3.3%)	4 (13.3%)
1.45–3.25 (Ishak fibrosis stages 2–3)	8 (26.7%)	7 (23.3%)
> 3.25 (Ishak fibrosis stages 4–6)	21 (70%	19 (63.3%)

Data presented as Mean ± SD unless otherwise are presented as %. AFP: Alpha-fetoprotein, ALT: Alanine aminotransferase, AST: Aspartate aminotransferase, INR: International normalized ratio, BCLC: Barcelona Clinic Liver Cancer staging system, FIB-4: Fibrosis-4 index, HCC: Hepatocellular carcinoma, HCV: Hepatitis C virus, and FIB4: Fibrosis-4 score.

### Diagnostic performance of biomarkers differentiating HCC from HCV-related cirrhosis patients

**Fig. 1:**
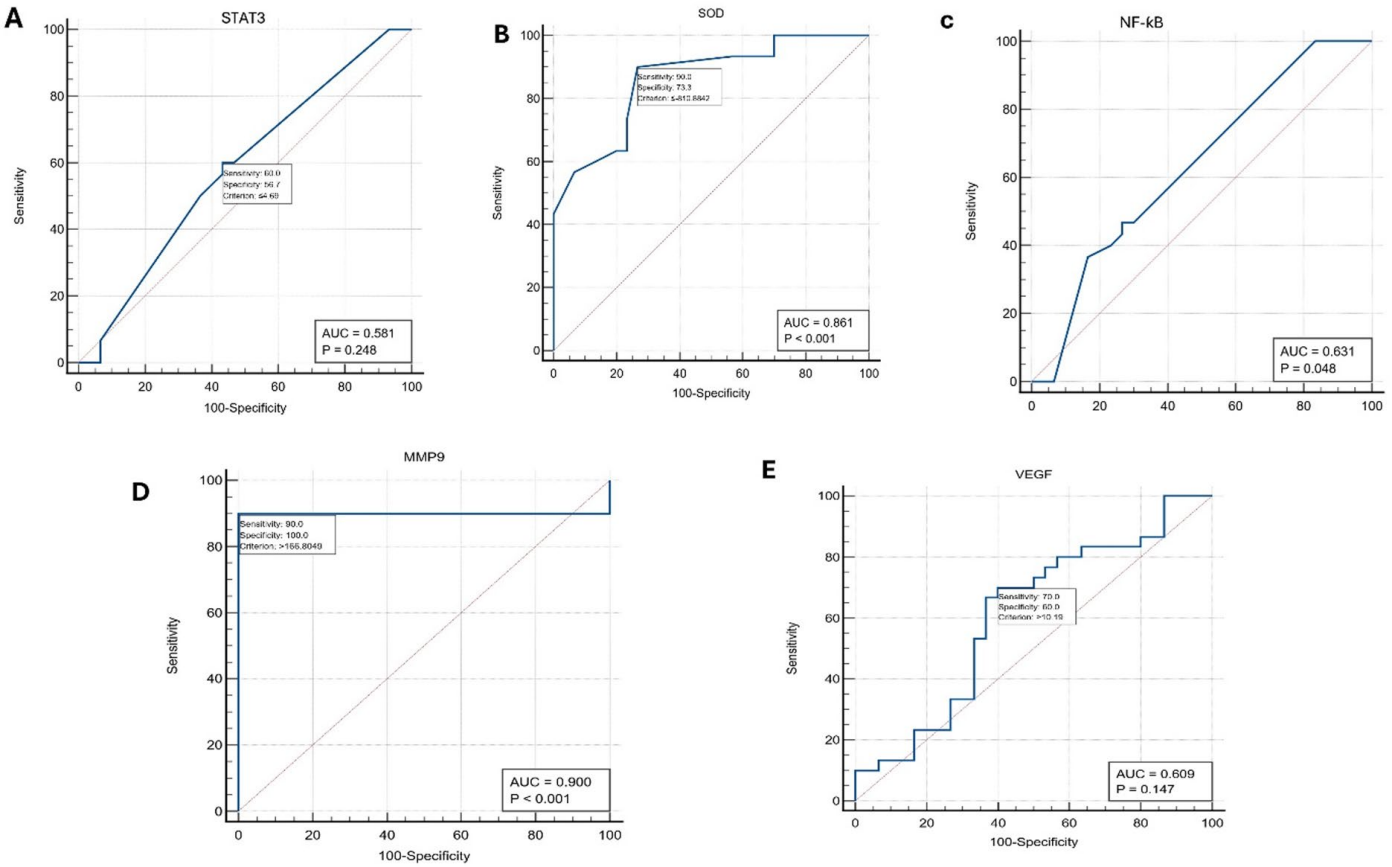
ROC curves of (**A**) STAT3, (**B**) SOD, (**C**) NF-κB, (**D**) MMP9, and (**E**) VEGF in distinguishing HCC from HCV- related cirrhosis. HCC, hepatocellular carcinoma, SOD, superoxide dismutase, MMP9, matrix metalloproteinase, VEGF; vascular endothelial growth factor, NF-κB, nuclear factor κ-B, and STAT3, Signal transducer and activator of transcription 3. SOD, superoxide dismutase, MMP9, matrix metalloproteinase, VEGF; vascular endothelial growth factor, NF-κB, nuclear factor κ-B, and STAT3, Signal transducer and activator of transcription 3.

**Fig. 2:**
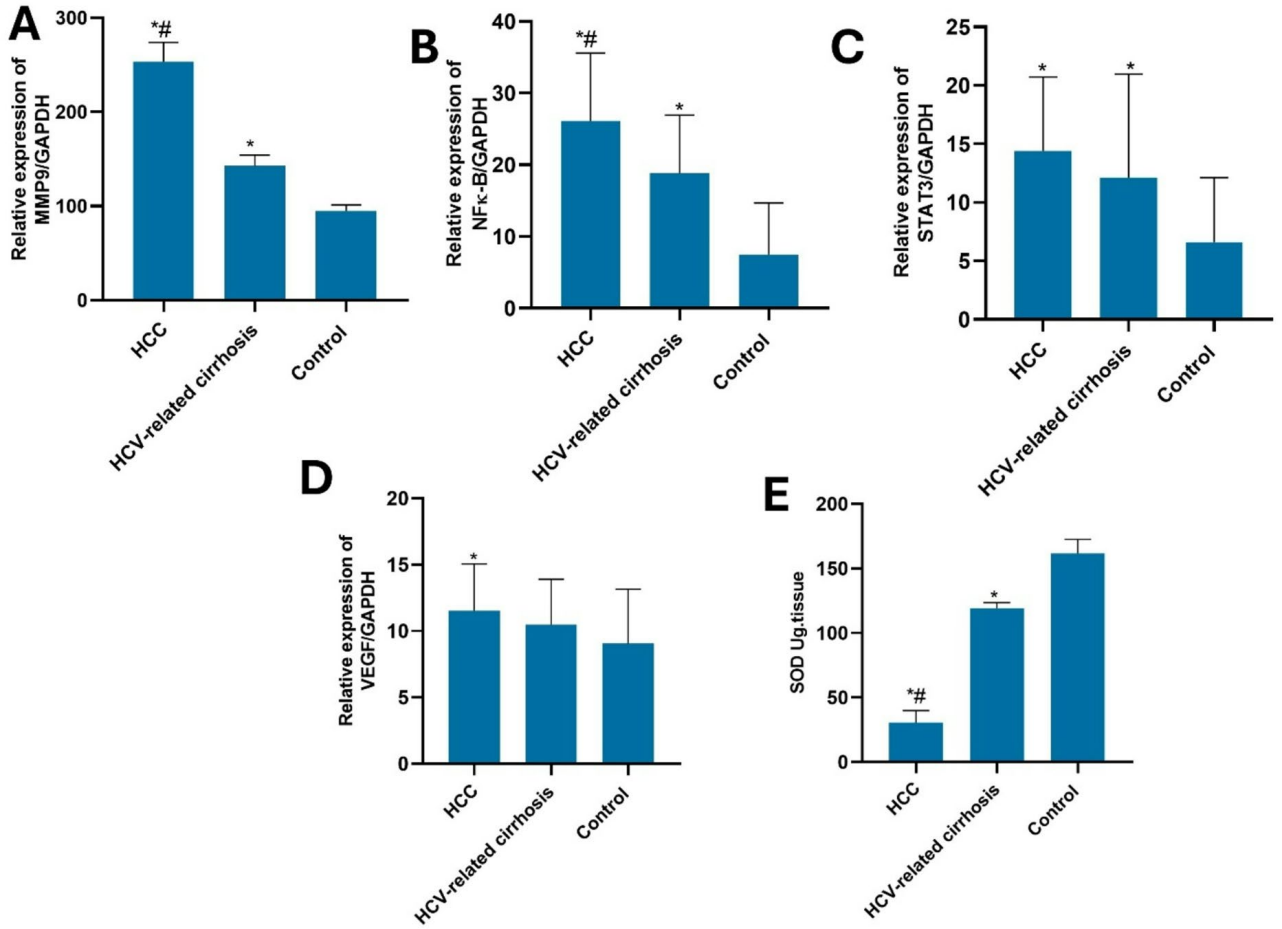
The relative expression of (**A**) MMP9, (**B**) NF-κB, (**C**) STAT3, and (**D**) VEGF and (**E**) the concentration of SOD in the blood samples of the studied group participants. * represents the significance against the control group and # represents the significance against the HCV group at *p* ≤ 0.05. MMP9; matrix metalloproteinase9, NF-κB; nuclear factor κ-B, STAT3; Signal transducer and activator of transcription 3, VEGF; vascular endothelial growth factor, SOD, superoxide dismutase, HCV; Hepatitis C virus, HCC, Hepatocellular carcinoma.

The Diagnostic performance of STAT3, SOD, NF-κB, VEGF, and MMP9 markers in diagnosing HCC vs. HCV was illustrated in Table 4; Figs. 1 and 2. The AUC for STAT3 was 0.581(95% CI: 0.446–0.797) and for VEGF was 0.609, which shows nonsignificant discriminators of HCC from HCV. On the other hand, the other 3 markers (SOD, NF-κB, and MMP9) were statistically significant discriminators of HCC from cirrhosis at cutoff values of ≤−810.8, ≤ 197, and > 166.8, respectively. Of the five biomarkers, MMP9 exhibited the highest diagnostic performance discriminating HCC vs. HCV. It showed the highest area under the curve value (0.9), and the highest specificity, sensitivity, negative predictive value (NPV), and positive predictive value (PPV). The specificity and sensitivity for each biomarker were 100% and 90% (for MMP9), 83.33% and 36.67% for NF-κB, 73.3% and 90% for SOD, 56.67% and 60% for STAT3, and 60% and 70% for VEGF, respectively. Furthermore, the PPV and NPV values for each marker are 100% and 90.9% for MMP9, 68.8% and 56.8% for NF-κB, 77.1% and 8% for SOD, 58.1% and 58.6% for STAT3, and 61.8% and 65.4% for VEGF, respectively.

Figure 2 also displayed that the relative expression of all mentioned markers was markedly elevated in the HCC group compared to the control group. The relative expression of MMP9, NF-κB, and SOD concentration also displayed a significant increase in HCV-related cirrhosis patients compared to the control group.

**Table 4:** Diagnostic performance of biomarkers in diagnosing HCC vs. HCV-related cirrhosis.

Biomarker	Cutoff	AUC	95% CI		Sig.	SN %	SP%	PPV%	NPV%
			Lower	Upper					
MMP9	> 166.8	0.9	0.795	0.962	< 0.0001	90	100	100	90.9
NF-κB	≤ 197	0.631	0.048	0.752	0.0479	36.7	83.3	68.8	56.8
SOD	≤−810.8	0.86	0.747	0.937	< 0.0001	90	73.3	77.1	88
VEGF	> 10.189	0.609	0.474	0.732	0.146	70	60	61.8	65.4
STAT3	≤ 4.69	0.581	0.446	0.797	0.248	60	56.7	58.1	58.6

Notes: AUC = Area under the ROC curve. CI = confidence interval. Sig. = Statistical significance (p-value). SP = specificity. SN = sensitivity. NPV = negative predictive value. PPV = positive predictive value.

.

### Distinct microbial compositions among control, HCV-related cirrhosis, and HCC groups

**Fig. 3:**
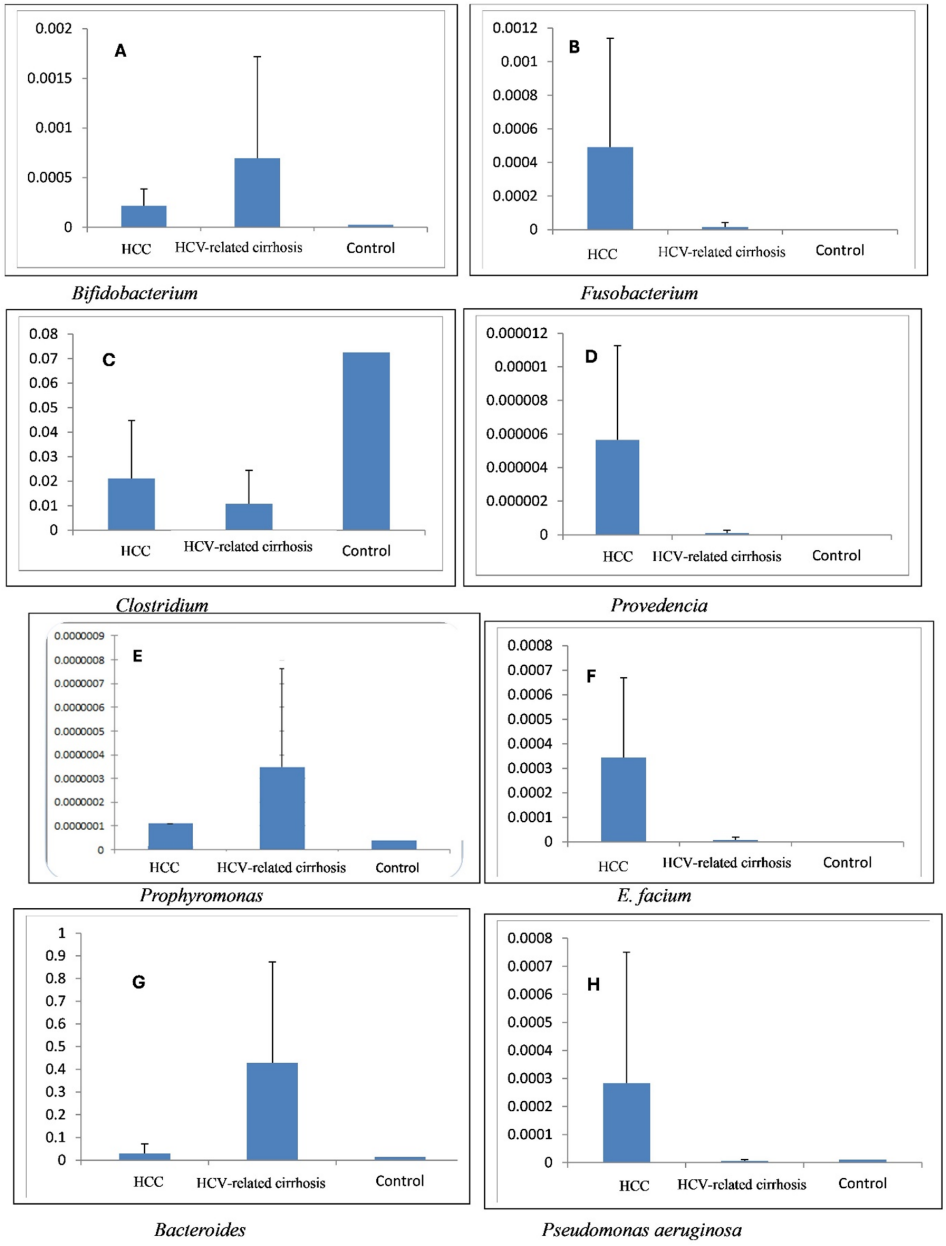
Relative abundance of diverse bacterial species in different tested sample groups measured through target genes specific to different reference microbiome relative to total bacteria count. (**A**) *Bifidobacterium spp*., (**B**) *Fusobacterium spp*., (**C**) *Clostridium spp*., (**D**) *Providencia spp*, (**E**) *Porphyromonas gingivalis*, (**F**) *E. faecium*, (**G**) *Bacteroides* spp and (**H**) *Pseudomonas aeruginosa*. HCC (hepatocellular carcinoma) and HCV (hepatitis C virus): represent diverse groups under test in this study.

To explore the microbial signatures in the HCC patients, we analyzed the expression of *Bacteroides spp.*,* Porphyromonas gingivalis*,* Bifidobacterium spp.*,* Enterococcus faecium*,* Fusobacterium spp.*,* Pseudomonas aeruginosa*,* Providencia spp.*,* and Clostridium spp.* in the fecal sample of HCC patients, HCV-related cirrhosis patients, and the control participants Fig. 3. Comparing the gene levels of HCV-related cirrhosis patients with those of healthy controls revealed that the most enriched genera in HCV-related cirrhosis patients were *Bifidobacterium*, *Porphyromonas*, and *Bacteroides*. Comparing the gene levels of HCC patients with those of healthy controls revealed that the most enriched genera in HCC patients were *Bifidobacterium*,* Fusobacterium*, *Providencia*,* E. faecium*,* and Pseudomonas aeruginosa.* Finally, by comparing the gene levels of HCC patients with those of HCV-related cirrhosis patients, the most enriched genera in HCC patients were *Fusobacterium*,* Providencia*, *E. faecium*, and *Pseudomonas aeruginosa.*

### Correlations between different Microbiome gene expression and biochemical indices

**Fig. 4:**
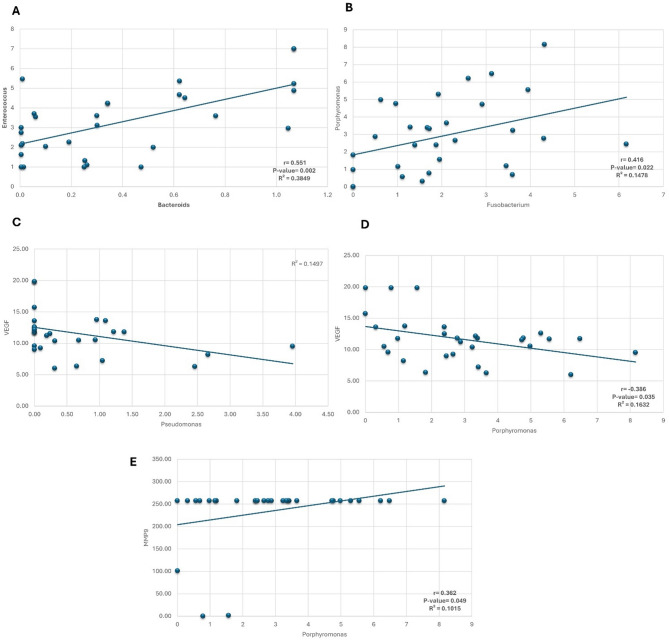
Correlations between different microbiome and biochemical markers. (**A**) Positive correlation between *Enterococcus* and *Bacteroides* (*r* = 0.551, *P* = 0.002). (**B**) Positive correlation between *Porphyromonas* and *Fusobacterium* (*r* = 0.416, *P* = 0.022). (**C**) Negative correlation between *Pseudomonas* and VEGF (*r* = −0.366, *P* = 0.047). (**D**) Negative correlation between *Porphyromonas* and VEGF (*r* = 0.416, *P* = 0.022). (**E**) Positive correlation between *Porphyromonas* and MMP9 (*r* = 0.362, *P* = 0.049).

Correlations between the different microbiome gene expressions and relevant biochemical indices were investigated (Tables 5 and 6**and** Fig. 4) to recognize the possible contributing impact of gut microbiomes in the pathogenesis of HCC. Five correlations were found to be significant. Our study revealed that *Enterococcus* and *Bacteroides* were positively correlated (*r* = 0.551, *P* = 0.002), Fig. 4A. *Porphyromonas* and *Fusobacterium* were also positively correlated (*r* = 0.416, *P* = 0.022), Fig. 4B. VEGF was negatively correlated with *Pseudomonas* (*r* = −0.366, *P* = 0.047), Fig. 4C and *Porphyromonas* (*r* = 0.386, *P* = 0.035), Fig. 4D. Conversely, there is a positive correlation between MMP9 and *Porphyromonas* (*r* = 0.362, *P* = 0.049), Fig. 4E.

**Table 5:** Correlation between the different Microbiome species.

		Clostridium	Bacteroides	Bifidobacterium	Fusobacterium	Providencia	Pseudomonas	Enterococcus	Porphyromonas
*Clostridium*	Correlation coefficient	1.000	0.119	0.167	0.064	−0.074	0.114	−0.001	−0.025
	Sig. (2-tailed)	0.697	0.529	0.378	0.736	0.697	0.547	0.995	0.897
*Bacteroides*	Correlation coefficient	0.119	1.000	−0.196	0.233	−0.297	0.050	**0.551** ^**^	0.361
	Sig. (2-tailed)	0.529	0.697	0.300	0.215	0.111	0.795	0.002	0.050
*Bifidobacterium*	Correlation coefficient	0.167	−0.196	1.000	−0.022	0.174	0.034	−0.025	−0.278
	Sig. (2-tailed)	0.378	0.300	0.697	0.906	0.359	0.860	0.895	0.136
*Fusobacterium*	Correlation coefficient	0.064	0.233	−0.022	1.000	−0.342	−0.059	−0.094	**0.416** ^*^
	Sig. (2-tailed)	0.736	0.215	0.906	0.697	0.064	0.756	0.620	0.022
*Providencia*	Correlation coefficient	−0.074	−0.297	0.174	−0.342	1.000	−0.059	0.020	−0.283
	Sig. (2-tailed)	0.697	0.111	0.359	0.064	0.697	0.757	0.915	0.129
*Pseudomonas*	Correlation coefficient	0.114	0.050	0.034	−0.059	−0.059	1.000	0.034	0.089
	Sig. (2-tailed)	0.547	0.795	0.860	0.756	0.757		0.860	0.640
*Enterococcus*	Correlation coefficient	−0.001	0.551^**^	−0.025	−0.094	0.020	0.034	1.000	−0.062
	Sig. (2-tailed)	0.995	0.002	0.895	0.620	0.915	0.860	0.697	0.743
*Porphyromonas*	Correlation coefficient	−0.025	0.361	−0.278	0.416^*^	−0.283	0.089	−0.062	1.000
	Sig. (2-tailed)	0.897	0.050	0.136	0.022	0.129	0.640	0.743	0.697

**. Correlation is significant at the 0.01 level (2-tailed).

*. Correlation is significant at the 0.05 level (2-tailed).

**Table 6:** Correlation between different Microbiome gene expression and biochemical markers.

		Age	SOD	STAT3	NF-κB	VEGF	MMP9	AFP
*Clostridium*	Correlation coefficient	−0.129	0.051	−0.002	−0.092	−0.192	0.212	0.025
	Sig. (2-tailed)	0.498	0.787	0.990	0.630	0.310	0.260	0.894
*Bacteroides*	Correlation coefficient	0.094	0.007	−0.005	0.106	−0.193	0.266	−0.097
	Sig. (2-tailed)	0.623	0.973	0.979	0.578	0.307	0.155	0.610
*Bifidobacterium*	Correlation coefficient	0.047	0.198	0.071	0.134	0.182	0.076	0.221
	Sig. (2-tailed)	0.804	0.294	0.709	0.480	0.336	0.688	0.241
*Fusobacterium*	Correlation coefficient	0.255	0.060	0.017	−0.062	−0.189	0.126	0.070
	Sig. (2-tailed)	0.174	0.752	0.928	0.747	0.318	0.507	0.715
*Providencia*	Correlation coefficient	0.167	−0.160	−0.264	0.261	0.180	−0.209	0.100
	Sig. (2-tailed)	0.379	0.400	0.158	0.163	0.340	0.267	0.600
*Pseudomonas*	Correlation coefficient	−0.249	−0.007	−0.118	0.329	− 0.366*	0.138	−0.058
	Sig. (2-tailed)	0.185	0.969	0.535	0.076	0.047	0.467	0.761
*Enterococcus*	Correlation coefficient	−0.056	0.170	0.129	0.220	−0.155	0.310	0.155
	Sig. (2-tailed)	0.770	0.370	0.498	0.243	0.415	0.096	0.414
*Porphyromonas*	Correlation coefficient	−0.004	0.053	−0.117	0.207	− 0.386*	0.362*	−0.299
	Sig. (2-tailed)	0.982	0.781	0.539	0.271	0.035	0.049	0.108

**. Correlation is significant at the 0.01 level (2-tailed).

*. Correlation is significant at the 0.05 level (2-tailed).

## Discussion

Hepatocellular carcinoma (HCC), the most frequently occurring liver cancer and the 4th cause of cancer-associated mortality globally, is correlated with an imbalance in microbiomes in the gut^34^. Alteration in the gut microbiome contributes to different diseases, including type 2 diabetes^35^, colorectal adenoma-carcinoma^36^, inflammatory bowel diseases^37^, and liver cirrhosis^38^. Different health problems are related to definite microbial profiles^39^. This study hypothesized that variation in the gut microbiome distribution, particularly between HCC and HCV-related cirrhosis participants, contributes to the development of HCC. Our experiment was conducted to establish a diagnostic model for HCC based on microbial markers, and firstly, we correlated these microbiomes with different diagnostic HCC biomarkers.

The gut microbiome has emerged as a hopeful means for identifying non-invasive biomarkers for various diseases, including cancer and metabolic disorders. Qin et al. illustrated the role of microbiomes as a diagnostic potential by identifying and validating 60,000 microbial markers associated with Type 2 Diabetes (T2D). They demonstrated that microbial profiles could be used effectively to classify T2D^35^. Another study done by Yu et al. conducted metagenomic profiling of fecal microbiomes from colorectal cancer (CRC) patients to identify microbiomes as biomarkers across ethnically diverse cohorts. Their findings highlight the potential for cost-effective biomarkers for early CRC diagnosis using fecal samples^40^. Another study also analyzed dysbiosis of the microbial gut in 98 liver cirrhotic patients and 83 healthy participants, identifying 15 microbial biomarkers that enabled accurate discrimination between patients and controls. This demonstrates the potential use of microbial biomarkers as an effective way of diagnosing liver cirrhosis^39^. In the current study, our selection of eight microbial taxa was guided by existing literature supporting their involvement in liver disease, particularly in HCV-related inflammation and hepatocarcinogenesis. For instance, *Fusobacterium nucleatum* has been shown to contribute to colorectal and liver cancer progression *via* activation of pro-inflammatory pathways^41^. *Bifidobacterium* species, while often associated with gut health, have also been implicated in promoting tumor-associated metabolic changes such as lactate accumulation^42^. Other selected taxa like *Bacteroides*,* Pseudomonas aeruginosa*, and *Enterococcus faecium* have demonstrated roles in promoting oxidative stress and inflammation, all of which are central to HCC development^43–45^.

Accordingly, 90 fecal samples from Egyptian participants were collected and analyzed using the real-time PCR technique allowed us to quantify these bacteria based on prior evidence of relevance specifically. Our study revealed that the relative expression of *Bifidobacterium*, *Porphyromonas*, and *Bacteroides* microbial species was markedly elevated in HCV-related cirrhosis patients compared to the healthy participants. The relative expression of *Bifidobacterium*,* Fusobacterium*, *Providencia*,* E. faecium*,* and Pseudomonas aeruginosa* was markedly elevated in the HCC patients, compared to the healthy participants. Furthermore, the expression of *Fusobacterium*,* Providencia*, *E. faecium*, and *Pseudomonas aeruginosa* showed higher abundance in HCC patients compared to the HCV-related cirrhosis group. These findings suggest that gut microbial biomarkers hold promise as a promising non-invasive way for early detection of HCC and liver inflammatory diseases like HCV.

*Bifidobacteria* are anaerobic, gram-positive, rod-shaped bacteria. They are primarily located in the mouth, cervix, colon, vagina, ileum, and occasionally in dental caries^46^. In ovarian cancer, an overabundance of *Bifidobacterium* was observed in cancer patients, compared to healthy participants^47^. These microorganisms were found to produce lactate, leading to elevated lactate levels^47^. This rise in lactate stimulates cell proliferation, angiogenesis, and metastasis, and also inhibits antitumor activity^48^. Similarly, our study revealed that the expression of *Bifidobacterium* in fecal samples of HCC patients is significantly higher than in healthy individuals. These observations underscore the context-dependent role of microbiota in cancer. While our study and others reported elevated *Bifidobacterium* abundance in HCC and HCV-related cirrhosis, some studies have observed decreased levels in rectal^49^ and pancreatic cancers^50^, suggesting that the influence of *Bifidobacterium* on tumor biology may differ according to cancer type and microenvironment.

*Fusobacterium nucleatum* is a Gram-negative bacterium present in the GIT and has been constantly recognized as a significant contributor to the incidence and progression of different cancers, such as pancreatic and colorectal cancer^51^. It facilitated the Polarization of M2 macrophage and increased the progression of colorectal carcinoma *via* inducing TLR4/NF-*κ*B/S100A9 Cascade. In agreement, our study also revealed that the expression of *Fusobacterium nucleatum* is markedly increased in HCC patient stools.

*Clostridium* spp. is an anaerobic, gram-positive bacterium found in the large intestine^52^. The toxigenic strains produce exotoxins, a pro-inflammatory protein, resulting in diarrhea and/or colitis^53^. In our study, *Clostridium* spp. were more abundant in the control group than in patients with HCV-related cirrhosis or HCC, consistent with their role as members of the commensal intestinal flora. the higher prevalence in healthy participants in our dataset suggests that not all *Clostridium* species are pathogenic, and their contribution to hepatocarcinogenesis likely depends on strain-specific and host-dependent factors. On the other hand, certain *Clostridium* strains have been implicated in carcinogenic or pro-inflammatory processes^54^. Previous study reported that the removal of *Clostridium* from thecultured bacterial community revealed that they were essential to transform the bacterial community from non-tumorigenic to tumorigenic^53^. *Clostridium* may act as a pro-mutagenic in the colon in mice^53^. Increased abundance of *Clostridium* was also detected in the gastric microbiota in gastric cancer patients in Taiwan^55^.

*Porphyromonas gingivalis* is an anaerobic organism and the primary periodontal infection. It was identified in the tumor region of esophageal cancer and correlated with cancer differentiation, TNM, and stage lymph node metastasis^56^. However, numerous studies have also shown the contributing role of *Porphyromonas* in different cancers, including esophageal squamous cell carcinoma^57^, colorectal carcinoma^58^, and gingival squamous cell carcinoma^59^. There is no previous study illustrated the role of *Porphyromonas gingivalis* in HCV-related cirrhosis and HCC. Our study revealed that the expression of *Porphyromonas gingivalis* is significantly higher in HCV-related cirrhosis patients than in normal participants. However, its expression in HCC patients is decreased compared to the HCV-related cirrhosis groups.

To elucidate the functional impact of microbial dysbiosis, we correlated microbial shifts with inflammatory and oxidative biomarkers. Oxidative stress is a prominent characteristic of hepatitis C infection. Although it is more pronounced in cirrhotic patients^60^. Additionally, oxidative stress is important in cancer development as the increase in ROS levels is mutagenic, as it could interact with the nitrogenous bases of DNA, forming DNA adducts^61^. Microbiome dysbiosis could increase oxidative stress due to the formylated peptides produced by commensal bacteria that could bind with macrophagic protein-coupled receptors, triggering an inflammatory cascade in epithelial cells. This process increases the production of superoxide, elevating cellular ROS^62^. Furthermore, the intestinal microbiome consumes glycine required for glutathione synthesis^63^. Our study revealed that the HCC and HCV-related cirrhosis groups with microbial dysbiosis displayed a marked decrease in the antioxidant SOD. Similarly, previous studies reported that *Bacteroides fragilis* and *Enterococcus faecalis* are associated with colorectal cancer and excess ROS production^64,65^.

Oxidative stress and inflammation are closely linked and mutually reinforcing. Nevertheless, several oxidants are generated by infiltrated inflammatory cells^66^. Activated monocytes and neutrophils elevate the production of •OH, H_2_O_2_, ONOO^−^, and O_2_^•−67^. Furthermore, ROS/RNS can also affect various pathways, including the NF-κB pathway, which induces the overexpression of several pro-inflammatory genes and subsequent promotion of several inflammatory cytokines^68^. In HCV-related cirrhosis, the transcription factors NF-κB and STAT3 are chronically activated, driving persistent inflammation, liver damage, and fibrosis^69^. This dysregulation helps HCV evade the immune system and promotes the progression from chronic hepatitis to cirrhosis and HCC^70^. Activated NF-κB promotes the release of several pro-inflammatory cytokines involved in angiogenesis, proliferation, apoptosis inhibition, and invasion^71^. STAT3 is another transcription factor activated by signaling pathways triggered by cytokines and growth factors^72^. Activated nuclear STAT3 has been identified in different cancers, including colon, breast, and HCC^73–75^. There is a connection between microbiomes and NF-κB activation, as bacterial lipopolysaccharides (LPS) are considered a chief promoter of NF-κB^76^. It was reported that the enteric microbiome contributes to inflammatory gut, as illustrated by the effective role of antibiotics administration in alleviating the inflammation in patients with inflammatory bowel disease^77^. Moreover, several colitis animal models illustrated that intestinal inflammation ameliorated when the animals were derived into a germ-free environment^78^. Our study showed that the expression of NF-κB and STAT3 in the blood samples of HCV-related cirrhosis and HCC groups with microbial dysbiosis is markedly higher than in the control participants. In agreement with our study, Gut dysbiosis exhibited a contributing role in prostate cancer development, progression, and docetaxel resistance through upregulating the NF-κB-IL6-STAT3 axis^79^.

Vascular endothelial growth factor (VEGF) is a vital inflammatory and angiogenesis factor, which is intimately correlated with several diseases, including psoriasis, autoimmune diseases, obesity, and tumors. VEGF is an important inducer of angiogenesis, and its expression is induced by several mediators, including growth factors and cytokines. While VEGF expression is detectable in non-tumoral hepatic parenchyma, significantly elevated levels are observed within tumor tissues^80^. In cancer, the levels of VEGF are elevated, and its expression is associated with poor prognosis^81^. The gut microbiome has been shown to contribute to the angiogenesis process and subsequently accelerate the spread of tumor cells^82^. The gut microbiomes generate several metabolites, which stimulate angiogenesis. Microbial LPS can promote the activation and upregulation of the VEGF^83^. Our study revealed a significant correlation between the expression of gut microbiome, like *Porphyromonas*, and the expression of VEGF. A previous study also explained the role of toxins from *Clostridium difficile* in inducing VEGF, influencing colonic vascular permeability, promoting disease pathogenesis^84^.

Besides VEGF, Matrix metalloproteinase 9 (MMP9) is a tumor angiogenic factor. It can also degrade components of the extracellular matrix and participate in tissue remodeling during the development of different conditions, including tissue repair, inflammation, tumor invasion, and metastasis^85^. The positive correlation between the expression of MMP9 and the abundance of gut microbiome in the current study indicates the significant role of gut microbiome in inducing tumor progression and invasion. Another study also showed the impact of the gut microbiome in metastasis, as *E. faecalis* was shown to activate MMP9 by activating the release of gelatinase E^86^.

By comparing the five diagnostic biomarkers of HCC, the present study demonstrated that MMP9 exhibited the best diagnostic marker, with the highest AUC at a cut-off value of > 166.8 and the highest SN, SP, PPV, and NPV. In agreement with our study, a recent study also revealed that ROC curve analysis of MMP9 proteins and gene expressions exhibited significantly marked specificity and sensitivity in diagnosing HCC than AFP^87^. Hayasaka et al. also showed that the ROC curve analysis of MMP9 levels in the plasma had a specificity of 89% and a sensitivity of 53% for distinguishing HCC at a 60 ng/mL cutoff value^88^. Furthermore, our study revealed that the plasma level of SOD also exhibited a significantly high AUC 0.86 at ≤−810.8 cut-off value with SN and SP 90% and 73.3%, respectively. Our SOD findings are consistent with another study reported that the cutoff value of SOD activity in detecting HCC in Chinese patients was 169.2 U/mL (SN: 87.23%, SP: 91.95%)^89^. Additionally, our findings revealed that NF-κB exhibited a significantly high AUC of 0.631 at a cut-off value ≤ 197 (SN = 36.67%, SP = 83.33%). On the other hand, the AUC for STAT3 and VEGF showed nonsignificant discriminators of HCC from cirrhosis in Egyptian patients. Together, our study added new insights to the growing evidence that microbial dysbiosis contributes not only to colorectal, pancreatic, and gastric cancers but also to HCC pathogenesis through shared oxidative stress, inflammation, metastasis, and angiogenesis pathways.

It is also important to note that these biomarkers exhibited differential expression between HCV-related cirrhosis and HCC, reflecting the progression of liver disease. In our cohort, MMP9, NF-κB, and SOD showed significant discriminative power between the two groups, indicating that oxidative stress and inflammatory pathways are already active in cirrhotic patients but become markedly amplified in HCC^90^. The elevated MMP9 expression in HCC compared to HCV-related cirrhosis underscores its role in extracellular matrix remodeling and tumor invasion, while the significant decline in SOD highlights increasing oxidative imbalance during malignant transformation. Conversely, NF-κB activation was evident in both conditions, supporting the thought that chronic inflammation in cirrhosis may be crucial in the hepatic microenvironment for tumorigenesis. These findings collectively suggest that monitoring these biomarkers could be valuable for distinguishing between advanced cirrhosis and early HCC development in HCV-infected patients.

Despite the promising findings, this study has some limitations. It was conducted at a single center with a relatively small cohort, which may introduce potential selection bias. Although participants were age- and sex-matched, unaccounted confounding factors such as diet and comorbidities could influence both microbiome composition and biomarker expression.

Future research involving larger, multi-center studies and shotgun metagenomic sequencing, uncovering a broader spectrum of gut dysbiosis in liver disease, is recommended to explore this potential. However, our targeted analysis provides a foundational framework for linking specific microbial shifts with biomarker dynamics in HCV-related cirrhosis and HCC. Additionally, Future studies are recommended to explore subgroup analyses for diabetic vs. non-diabetic HCV patients and HCV-HCC vs. non-HCV HCC, and the role of metabolic factors in HCC progression more comprehensively.

## Conclusion

This study exhibited the presence of characteristic intestinal microbiome dysbiosis with significant enrichment of *Bifidobacterium*,* Fusobacterium*, *Providencia*,* E. faecium*,* and Pseudomonas aeruginosa* species in HCC patients. On the other hand, our study revealed that the relative expression of *Bifidobacterium*, *Porphyromonas*, and *Bacteroides* microbial species was significantly increased in HCV-related cirrhosis patients compared to the healthy participants. Our study also explored the role of SOD, MMP9, and NF-κB as diagnostic differentiators between HCV-related cirrhosis and HCC.

## Data Availability

Data are available from the corresponding author on reasonable request.
